# Improvement of Oxidative Stress and Mitochondrial Dysfunction by *β*-Caryophyllene: A Focus on the Nervous System

**DOI:** 10.3390/antiox10040546

**Published:** 2021-04-01

**Authors:** Hammad Ullah, Alessandro Di Minno, Cristina Santarcangelo, Haroon Khan, Maria Daglia

**Affiliations:** 1 Department of Pharmacy, University of Naples Federico II, 80131 Naples, Italy; hammad.ullah@unina.it (H.U.); alessandro.diminno@unina.it (A.D.M.); cristina.santarcangelo@unina.it (C.S.); 2 CEINGE-Biotecnologie Avanzate, 80131 Naples, Italy; 3 Department of Pharmacy, Abdul Wali Khan University, Mardan 23200, Pakistan; hkdr2006@gmail.com or; 4 International Research Center for Food Nutrition and Safety, Jiangsu University, Zhenjiang 212013, China

**Keywords:** oxidative stress, mitochondrial dysfunction, neurodegeneration, *β*-caryophyllene, neuroprotection

## Abstract

Mitochondrial dysfunction results in a series of defective cellular events, including decreased adenosine triphosphate (ATP) production, enhanced reactive oxygen species (ROS) output, and altered proteastasis and cellular quality control. An enhanced output of ROS may damage mitochondrial components, such as mitochondrial DNA and elements of the electron transport chain, resulting in the loss of proper electrochemical gradient across the mitochondrial inner membrane and an ensuing shutdown of mitochondrial energy production. Neurons have an increased demand for ATP and oxygen, and thus are more prone to damage induced by mitochondrial dysfunction. Mitochondrial dysfunction, damaged electron transport chains, altered membrane permeability and Ca^2+^ homeostasis, and impaired mitochondrial defense systems induced by oxidative stress, are pathological changes involved in neurodegenerative disorders. A growing body of evidence suggests that the use of antioxidants could stabilize mitochondria and thus may be suitable for preventing neuronal loss. Numerous natural products exhibit the potential to counter oxidative stress and mitochondrial dysfunction; however, science is still looking for a breakthrough in the treatment of neurodegenerative disorders. *β*-caryophyllene is a bicyclic sesquiterpene, and an active principle of essential oils derived from a large number of spices and food plants. As a selective cannabinoid receptor 2 (CB2) agonist, several studies have reported it as possessing numerous pharmacological activities such as antibacterial (e.g., *Helicobacter pylori*), antioxidant, anti-inflammatory, analgesic (e.g., neuropathic pain), anti-neurodegenerative and anticancer properties. The present review mainly focuses on the potential of *β*-caryophyllene in reducing oxidative stress and mitochondrial dysfunction, and its possible links with neuroprotection.

## 1. Introduction

Mitochondrial dysfunction refers to an impairment in mitochondrial function, resulting in a series of defective cellular events including decreased adenosine triphosphate (ATP) production, enhanced reactive oxygen species (ROS) output, altered proteostasis and cellular quality control [1]. Neurons have increased demand for ATP and oxygen, with cortical neurons being known to consume approximately 4.7 billion ATP molecules per second, and thus they are more prone to the damage induced by mitochondrial dysfunction [2]. Such critical requirements for ATP and oxygen make them susceptible to electron leakage from the electron transport chain, resulting in generation of free radicals and induced oxidative stress [3]. Furthermore, lowered levels of antioxidant defenses further increase the neuronal susceptibility to mitochondria induced oxidative damage [4]. In addition, a large ROS output not only damages the biomolecules of the neuronal cells, but also damages mitochondrial components (e.g., mitochondrial DNA) and elements of the electron transport chain, resulting in a loss of electrochemical gradient across the mitochondrial inner membrane and the ensuing shutdown of mitochondrial energy production [5].

The electron transport chain, or mitochondrial respiratory chain, consists of five complexes (complex I, II, III, IV and V) and is one of the major structural and functional components of mitochondria, catalyzing the phosphorylation of adenosine diphosphate (ADP) to ATP [6]. These complexes are comprised of over 80 proteins, 13 of which are encoded by mitochondrial DNA and are components of oxidative phosphorylation [6,7]. Complexes I–IV constitute the electron transport chain, which generates water by oxidation of hydrogen (derived from organic acids like pyruvic and fatty acids) with atomic oxygen [8]. ATP production involves two coordinated processes, including transport of electrons along the complexes to produce water and the pumping of protons across the mitochondrial inner membrane (from matrix to intermembrane space) through complexes I, III and IV. ATP is thus generated by the influx of these protons back to the matrix through complex V [9,10,11]. Under normal physiological conditions, 1.5% of the oxygen may be converted into ROS, which suggests that the majority of intracellular ROS is generated by mitochondria [12]. The production of superoxide and other reactive oxygen species occurs primarily at complexes I and III [13]. Under pathological conditions, the highly reactive hydroxyl ions could damage mitochondrial DNA, proteins, and lipids, resulting in the defective functioning of complexes I and III, causing superoxide radical formation by increased electron reduction of oxygen, leading to metabolic oxidative stress, genomic instability, and cellular injury [14,15,16,17].

Damaged mitochondrial DNA may decrease the expression of critical proteins of the electron transport chain, amplifying oxidative stress which eventually triggers apoptosis [14]. The electron transport chain is also sensitive to nitrosative stress, as nitration can modify mitochondrial proteins, causing alterations in the functioning of many metabolic enzymes in the electron transport chain, such as nicotinamide adenine dinucleotide (NAD) dehydrogenase, cytochrome c oxidase, and ATP synthase [18]. Most importantly, acute exposure to ROS inactivates the iron-sulfur centers of complexes I, II and III, while chronic exposure can damage cellular and mitochondrial proteins, lipids, and genetic materials [7]. ROS also alters mitochondrial membrane permeability, as the inner membrane is located near the site of ROS production and thus is more prone to lipid peroxidation [19]. Peroxidation of mitochondrial phospholipids may increase the proton permeability of the inner membrane, which under normal physiological conditions is permeable only to tiny neutral molecules [20]. Increased membrane permeability could lead to altered fluidity, as well as impaired biochemical functions of numerous transporters and enzymes present in mitochondrial membranes [12].

**Figure 1:**
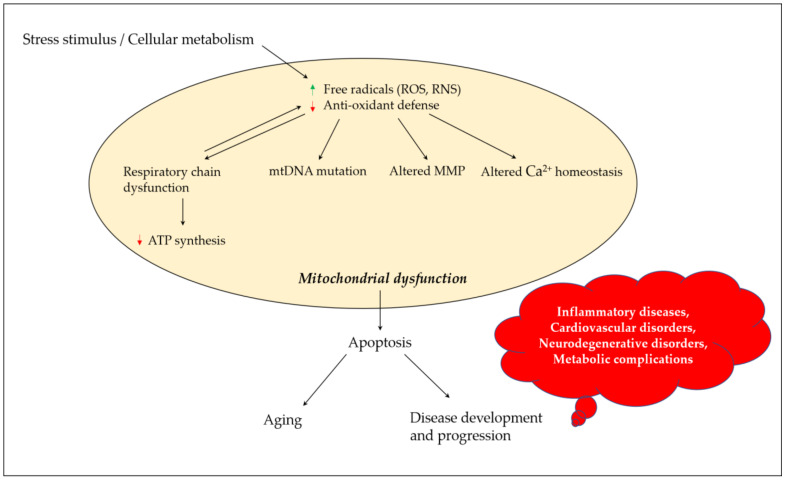
Mitochondrial dysfunction and its contribution towards aging and disease development and progression. Stress stimulus and irregular cellular metabolism may lead to the increased production of ROS and RNS, and decreased antioxidant defense parameters, which eventually result in mitochondrial dysfunction due to a defective mitochondrial respiratory chain, mutation in mtDNA, altered MMP and influenced Ca^2+^ homeostasis. These events could promote apoptosis, paving the road for aging and disease development and progression. ROS, reactive oxygen species; RNS, reactive nitrogen species; mtDNA, mitochondrial DNA; MMP, mitochondrial membrane potential; ATP, adenosine triphosphate.

Mitochondria play a critical role in regulating neuronal Ca^2+^ homeostasis, and genetic and pharmacologic manipulations enhancing mitochondrial Ca^2+^ sequestration may protect neuronal cells against excitotoxicity [21]. Excessive ROS generation alters mitochondrial Ca^2+^ homeostasis, where peroxynitrite inactivates key mitochondrial enzymes, affecting the energy status of the cell and triggering the release of Ca^2+^ from the mitochondria [22]. Elevated Ca^2+^ levels cause a shift in mitochondrial potential and result in the production of superoxide radicals which may lead to a vicious cycle. Changes in mitochondrial permeability in Ca^2+^ overloaded mitochondria result in osmotic swelling and the rupture of the outer mitochondrial membrane [23]. ROS production in mitochondria further promotes Ca^2+^ uptake and enhances membrane permeability, and eventually results in the release of cytochrome c and the initiation of apoptosis [24]. Figure 1 depicts the links between oxidative stress and mitochondrial dysfunction, and their possible impact on aging and disease development and progression.

Many antioxidant agents were found to be active when tested in animal models of neurodegeneration (e.g., vitamin E), but unfortunately showed no or little benefits in clinical settings. Identifying bioactive substances that can counter oxidative damage as well as restore mitochondrial dysfunction may be a fruitful approach in reversing neurodegeneration [3] The present review is designed to highlight the potential of *β*-caryophyllene in reducing oxidative stress and mitochondrial dysfunction, with a special focus on the nervous system.

## 2. Chemistry and Vegetable Sources of *β*-Caryophyllene

**Figure 2:**
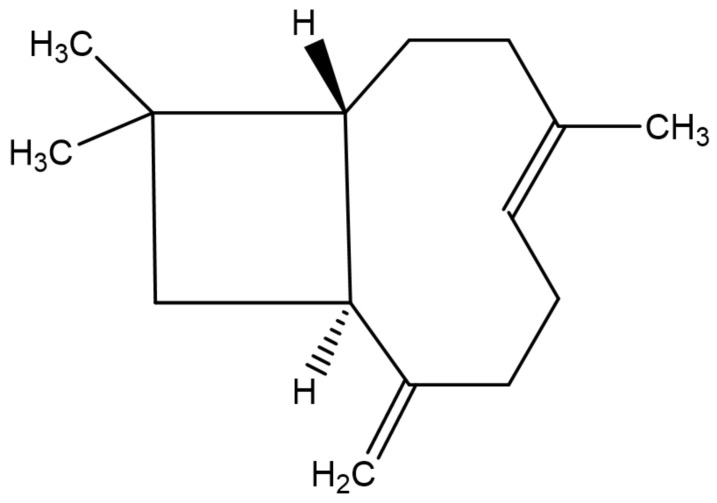
Chemical structure of *β*-caryophyllene.

*β*-caryophyllene (Figure 2) is a bicyclic sesquiterpene, mainly occurring in the form of trans-caryophyllene in combination with small amounts of its isomers (iso-caryophyllene and α-caryophyllene or α-humulene) and its oxidative derivative *β*-caryophyllene oxide. *β*-caryophyllene and *β*-caryophyllene oxide are compounds with a strong wooden odor and are approved as flavorings by the Food and Drug Administration (FDA) and European Food Safety Authority (EFSA) [25]. *β*-caryophyllene exhibits low water solubility and thus aqueous media such as biological fluids decrease its absorption to the cell. However, the potential obstacles associated with its low water solubility can be overcome by liposomal formulation techniques, which could increase its bioavailability and ensure the desired biological cell effects [26]. *β*-caryophyllene is a major active principle of essential oils derived from a large number of spices and food plants (Table 1). As reported in the Essential Oil Database, in nature *β*-caryophyllene is commonly found in *Ocimum basilicum* L., *Cinnamomum* species, *Piper nigrum* L., *Syzygium aromaticum* (L.) Merr. and L.M. Perry, *Cannabis sativa* L., *Lavandula angustifolia* Mill., *Origanum vulgare* L., and *Rosmarinus officinalis* L. [27].

## 3. Biological Activities of *β*-Caryophyllene

*β*-caryophyllene is a selective cannabinoid type 2 (CB2) receptor agonist, and a number of studies have reported its pharmacological activities, such as antibacterial [28], anti-*Helicobacter pylori* [29], antioxidant [30], anti-inflammatory [30], analgesic [25], neuroprotective [31], and anticancer potentials [25]. Cannabinoids have gained increased scientific attention throughout recent decades, due to their range of biological activities across the body. Their biological impact is exerted through cannabinoid type 1 (CB1) and CB2 receptors. CB1 receptors are widely distributed in the brain, and cannabinoids impart their psychoactive effects via these receptors, while CB2 receptors have a more restricted distribution with the majority being in immune cells and only a few in the brain. Moreover, both these receptors are coupled with inhibitory G-proteins (Gi) and are subjected to same pharmacological influences as other G-protein coupled receptors [32]. The primary action of CB2 receptor ligands is in the inhibition of inflammatory pathways, and they can thus be utilized in the treatment of inflammatory conditions such as osteoporosis, osteoarthritis, colitis, and atherosclerosis [33]. 

## 4. Mitochondrial Dysfunction and Neurodegeneration

Both oxidative stress and mitochondrial dysfunction are essential hallmarks of the early pathological mechanisms of aging and neurodegenerative disorders, i.e., Alzheimer’s disease (AD), Parkinson’s disease (PD), Multiple sclerosis (MS), amyotrophic lateral sclerosis (ALS) and Huntington’s disease (HD). Mitochondrial dysfunction, damaged electron transport chains, altered membrane permeability and Ca^2+^ homeostasis, and impaired mitochondrial defense systems induced by oxidative stress are pathological changes implicated in neurodegenerative disorders by amplifying neuronal dysfunction or triggering neurodegeneration [34]. The involvement of oxidative stress or mitochondrial dysfunction within individual neurodegenerative disorders has been described below.

### 4.1. Alzheimer’s Disease (AD)

AD mostly affects the elder population (aged 65 years or older) and contributes towards 65–80% of total dementia cases [35]. Its central pathogenic mechanisms are the accumulation of amyloid-*β* (A*β*) aggregates and hyperphosphorylation of tau proteins, resulting in neurofibrillary tangles (NFTs) and synaptic dysfunction [36,37,38]. Increased ROS generation could trigger A*β* aggregation early in the course of the disease, aiding in disease initiation and progression. Oxidative changes to A*β* proteins may lead to protein misfolding and aggregation. Alteration in the phosphorylation of proteins (biliverdin reductase A and heme oxygenase-1) may affect the signaling of the most crucial antioxidant pathways. This results in mitochondrial damage which in turn stimulates the generation of high levels of ROS, against which antioxidant defenses may be deficient [39]. In addition, altered activity of the α-ketoglutarate dehydrogenase enzyme complex, which mediates the oxidative metabolism, has been noted in brains from Alzheimer’s patients [40]. Deficiency of mitochondrial dihydrolipoyl succinyltransferase enzyme (one of the key subunits specific to α-ketoglutarate dehydrogenase enzyme complex activity) has been found to increase A*β* aggregation and nitrotyrosine levels in female Tg19959 mice [41]. In late-onset AD mitochondria also generate age related free radicals, which are carried to cytoplasm where they upregulate *β*-secretase and accelerate the cleavage of amyloid precursor protein molecules, which further enhance the production of free radicals and result in the disruption of electron transport chain and enzyme activities [40,42,43].

Unlike in PD, the role of mitochondria in the pathogenesis of AD is still disputed and its genetic basis remains largely unknown. Mutation in the genes for Amyloid Precursor Protein (*APP*), Presenilin 1 (*PS1*) and Presenilin 2 (*PS2*) have been implicated in early-onset AD, their functions being in processing APP polypeptide. APP is cleaved into A*β* peptides (A*β*_1–40_ and A*β*_1–42_) when processed by *β* and γ- secretases [44,45]. The PS complex proteins have been found in mitochondria, suggesting their potential role in normal mitochondrial function, and mutations in PS complex proteins may thus enhance sensitization of cells to apoptotic stimuli at the mitochondrial level [46]. Moreover, PS together with nicastrin, anterior pharynx-defective-1 (APH-1), and presenilin enhancer-2 (PEN-2), forms a catalytic core for the γ-secretase protein complex in mitochondria [46,47]. The importance of PS for secretase activity has been demonstrated in different ways (i.e., (a) total inactivation of γ-secretase in PS-deficient embryonic cells [48], and (b) the use of PS binding γ-secretase inhibitors) [49,50]. These studies resulted in downstream regulation of γ-secretase activity and an eventual decrease in A*β* production. 

PS located in mitochondrial membranes may possess a crucial role in the opening of megapores during permeability transitions or cytochrome c release, and thus mutations in PS1 could facilitate such processes, making cells more vulnerable to apoptosis at the mitochondrial level. As PS1 is part of the γ-secretase protein complex in mitochondria, such activity may cleave and activate proteins that may be involved in the initiation of apoptosis [46,51]. Interestingly, the true substrate for mitochondrial γ-secretase is yet to determined, as *β*-APP is not a substrate for γ-secretase activity in mitochondria, where γ-secretase also cleaves several other type 1 transmembrane proteins, and such protease activity may contribute to other neurodegenerative pathways beyond A*β* generation and plaque formation [46].

### 4.2. Parkinson’s Disease (PD)

The neuropathological mechanisms of PD involve the misfolding of proteins, disrupted protein handling, neuroinflammation, oxidative stress, mitochondrial dysfunction and impaired Ca^2+^ handling [52]. The theory of mitochondrial dysfunction in PD came from studies in which the accidental infusion of 1-methyl-4-phenyl-1, 2, 3, 6-tetrahydrodropyridine (MPTP), a byproduct found in synthetic heroin, selectively inhibited the mitochondrial complex I [53,54]. Other inhibitors of complex I include pyridaben, rotenone, fenpyroximate, and trichloroethylene, which have been reported to induce dopaminergic neurodegeneration in rodents, flies, and humans [55]. Impairments in the activity of complex I have been observed in the substantia nigra, skeletal muscles and platelets of PD patients [55,56,57]. Structural modifications in complex I (resulting from deficiency in apoptosis-inducing factor) actually do not lead to neurodegeneration but increase the sensitivity of dopaminergic neurons to neurotoxins [58].

Mutations or polymorphisms in mitochondrial DNA or nuclear genes (α-synuclein, tau, parkin, C-terminal hydrolase-L1, ubiquitin DJ-1, leucine-rich-repeat kinase 2, PTEN (phosphatase and tensin homologue)-induced kinase 1, nuclear receptor NURR1, and HTRA2) have been identified as possible risk factors for developing PD, where nuclear genes α-synuclein, DJ-1, parkin, phosphatase and tensin homologue-induced kinase 1, leucine-rich-repeat kinase 2, and HTRA2 involve mitochondria [34]. Mutations in parkin is mostly responsible for early onset familial PD [59]. Parkin and PTEN (phosphatase and tensin homologue)-induced kinase 1 (PINK1) are functionally correlated, as their expression promotes mitochondrial fission [60,61]. Downregulation of PINK1 may lead to reduced mitochondrial protection against oxidative stress, whereas parkin is recruited to defective mitochondria to promote their autophagic degradation and to rescue degeneration in PINK1 [62,63]. 

Regulation of stress-mediated mitochondrial quality control by *parkin* and *PINK1* has become more evident with more scientific research into mitochondria-associated degradation, mitochondria-derived vesicles, and organelle biogenesis. *PINK1/parkin* is a complex regulated, sequential process that modifies a wide range of substrate proteins and mediates their clearance, including mitochondrial fusion proteins (mitofusin-1 and mitofusin-2) and outer mitochondrial membrane proteins (miro-1 and miro-2) [64]. Mitofusins could be targeted to prevent refusion of impaired mitochondria within healthy networks, and these are eliminated by valosin-containing protein via mitochondria-associated degradation by 26 S proteosome [65,66]. Outer mitochondrial membrane proteins are involved in mitochondrial dynamics by anchoring mitochondria to microtubules through the kinesin motor protein, playing a considerable role in their trafficking. Degradation of these transporter proteins could result in the suppression of mitochondrial movement, further promoting the segregation of damaged mitochondria [67].

Mutations in *DJ-1* (mitochondrial peroxiredoxin-like peroxidase) cause an autosomal recessive form of PD, where *DJ-1* is known to scavenge mitochondrial radical species [68]. In vivo studies showed that mice with overexpression of *α-synuclein* were more susceptible to the degenerative effects induced by MPTP while *α-synuclein* knockout mice were found to be protected against the neurotoxic impacts of MPTP, 3-nitropropionic acid (3-NP), and malonate [69,70].

### 4.3. Multiple Sclerosis (MS)

MS is a progressive neuromuscular disorder, characterized by an autoimmune attack targeting the myelin sheath of nerve cell axons in the brain and spinal cord, which leads to demyelination of neurons [71,72]. Pathogenesis of the disease usually begins at an age range of 20–50 years, with women usually being more susceptible than men as the disease is X-chromosome dependent [2,73]. The actual cause of MS initiation and progression are yet to be established, but studies have shown that iron overload mediated production of protease and glutamate and generation of reactive oxygen and nitrogen species may promote breakdown of the myelin sheath [74]. Activation of microglia is the main event occurring early in the course of the disease, releasing nitric oxide (NO) and increasing production of major excitatory neurotransmitter glutamate, which leads to injury of nerve fibers from targeting of intracellular Na^+^ and Ca^2+^ concentrations. This eventually results in transitions in membrane permeability, mitochondrial swelling, and rupture of the mitochondrial membrane. The reactive oxygen and nitrogen species may lead to apoptosis via the cytochrome c pathway and an impaired electron transport chain, respectively [75]. The process is coordinated with the infiltration of inflammatory cytokines, which cause progressive loss of nerve fibers and contribute to neurological impairment in patients with MS [2].

### 4.4. Amyotrophic Lateral Sclerosis (ALS)

ALS is described by gradual muscle atrophy, weakness, and respiratory failure, characterized by a progressive loss of motor neurons in the anterior horn of the brain and spinal cord [76]. In 5% of cases, ALS is caused by a mutation in genes including *TARDBP*, *SOD1* (cytosolic SOD), *FUS*, *C9orf72*, *TAF15* and *UBQLN2*. Mutations in *SOD1*, increased oxidative and nitrosative stress and dramatic gliosis (aberrant astrocytes, extensive astrocytosis, upregulated inducible nitric oxide synthase (iNOS) expression and activated microglial cells) are some of the pathologic changes noted in ALS [77]. *SOD1* mutation is not only associated with morphological changes in mitochondria but also linked with mitochondrial dysfunction [78]. Additionally, mutations in mitochondrial DNA, mitochondrial transfer RNA and cytochrome c have also been noticed in ALS patients [79]. Transgenic mice expressing SOD1 mutants G37R or G93A in their motor neurons showed massive mitochondrial swelling and vacuolation, which appeared to be derived from degenerated mitochondria [80,81]. 

Since *SOD1* mutation accounts for only around 20% of familial cases of ALS [82], other proteins associated with the pathogenesis of ALS are still to be discovered. Two recently identified essential hallmarks of ALS characterized by the presence of ubiquitin-positive inclusions (FTLD-U) are TAR DNA binding protein-43 (TDP-43) and Fused-in-Sarcoma (*FUS*) protein. These proteins are normally located in the nucleus but pathological TDP-43 and *FUS* inclusions can be predominantly found in the cytosol [83,84,85,86,87]. TDP-43 have well-organized roles in the regulation of transcription and splicing, microRNA processing, stabilization of mRNA, apoptosis, and cell death, whereas *FUS* is implicated in number of cellular processes such as cell proliferation, DNA repair, transcription regulation, and microRNA processing [82].

*TDP-43* gene mutations have been associated with TDP-43 proteinopathies such as ALS, characterized by the presence of inclusions composed of abnormal TDP-43 [88]. Electron microscopy analysis of brain samples of TDP-43 proteinopathy patients revealed prominent mitochondrial impairment, with the results being consistent with cellular and animal models [89]. In these models, induced mitochondrial dysfunction, including decreased mitochondrial membrane potential (MMP) and elevated ROS production, was observed with increased TDP-43 expression. Suppressed mitochondrial complex I activity, reduced mitochondrial ATP synthesis and upregulated mitochondrial unfolded protein response were also correlated with TDP-43 expression. Additionally, downstream regulation of mitochondrial protease LonP1 was associated with enhanced mitochondrial TDP-43 levels and thus aggravated TDP-43 induced mitochondrial impairment as well as neurodegeneration [89]. 

*FUS* gene mutations can be linked with familial ALS, displaying *FUS*-positive inclusions [90], while the overexpression of ALS-mutant *FUS* may led to progressive neurodegeneration, reiterating findings in patients [90,91]. Numerous studies implicated mitochondrial damage as an early event preceding cell death in *FUS* proteinopathies [92,93]. Deng et al. observed the reduced MMP and increased mitochondrial ROS production with an overexpression of mutant *FUS* in HEK293 cells [92]. Enhanced ROS levels may drive mitochondrial translocation of the pro-fission protein DRP1 in ASTCa1 cells, resulting in mitochondrial fragmentation [94]. Likewise, Deng et al. noted mitochondrial fragmentation with overexpression of mutant *FUS* genes in HT22 cells, cultured neurons, and transgenic fly motor neurons [92]. Electron microscopy of these cells displayed a marked loss or disruption of cristae with frequent detection of “onion-like” deformed shapes.

### 4.5. Huntington’s Disease (HD)

HD is a neurodegenerative disorder affecting muscle coordination and the decline of mental states, and is linked with repetitive expansion of cytosine, adenine, and guanine (CAG) in the huntingtin (*HTT*) gene, resulting in elongated polyglutamine stretch and development of *HTT* protein product, which aggregates throughout the brain [95]. Oxidative stress is the main factor provoking pathogenesis of HD, as reactive oxygen species contribute to protein misfolding causing the formation of inclusion bodies that clump together at axons and dendrites in neurons and stop neurotransmission [96]. Mitochondrial DNA has been suggested as a main target for HD associated oxidative stress, and the activity of complex II has been observed to decrease [56,97]. Biochemical analysis of brain tissues from HD patients revealed the impaired activity of electron transport chain complexes and tricarboxylic acid cycle enzymes, which could result in reduced mitochondrial biogenesis, ATP deficit, oxidative stress, and elevated apoptosis. The activity of tricarboxylic acid cycle enzyme aconitase is particularly regulated by reactive oxygen and nitrogen species, and other toxic molecules [95]. Moreover, accumulation of oxidative stress markers such as lipofuscin (derived from peroxidation of unsaturated fatty acids), protein oxidation and nitrosylation, increased iron metabolism, and 8-hydroxy-2-deoxyguanosine in mitochondria are well-described features of HD brains [95].

### 4.6. Other Neurological Disorders

Friedreich’s ataxia (FRDA) is one the most common hereditary ataxias in the Caucasian population, caused by triplet extensions in the *frataxin* gene, characterized by iron accumulation in mitochondria, resulting in impaired activities of complex I–III, which lead to oxidative stress and accumulation of free radicals. The depletion of *frataxin* in mitochondria could be a consequence of FRDA, as *frataxin* regulates mitochondrial handling of iron and thus protects against damage mediated by iron-induced oxidative stress [74]. Transmissible spongiform encephalopathies (TSEs) such as Creutzfeldt–Jakob disease, Kuru, bovine spongiform encephalopathy (BSE), fatal familial insomnia, and Gerstmann–Straussler–Scheinker syndrome are lethal neurodegenerative pathologies, caused by misfolding and aggregation of prion proteins. Occurrence of TSEs is linked with oxidative stress-induced formation of prion proteins [98]. 

## 5. Mitochondria-Based Therapies in Neurodegenerative Disorders

Many antioxidant compounds have been identified as maintaining redox balance in chronic degenerative disorders, but none of them can halt the progression or cure the disease. Scientists have turned their attention to breakthroughs in the treatment of neurodegenerative disorders by focusing more on agents targeting mitochondria [3], however the pre-clinical and clinical data regarding these agents is still very limited in experimental models of these diseases. Health friendly agents in this context that may reverse oxidative damage include creatine, coenzyme Q (CoQ10), *Ginkgo biloba* L., remacemide, riluzole, α-lipoic acid and essential fatty acids [99,100]. 

Creatine is commonly found in meat and fish products and is a potent stimulator of the mitochondrial respiratory chain, by the generation of phospho-creatine which is known to be an essential component of the creatine kinase (CK) system in the maintenance of cellular energy needs, as it aids in production of ATP molecules by transferring the phosphoryl group to ADP in the presence of enzyme creatine kinase, maintaining a balanced energy homeostasis. Literature data have suggested a potential role for creatine supplementation in mitochondrial encephalomyopathies, cerebral ischemia and stroke, and traumatic injuries of CNS, AD, PD, ALS, and HD [101,102]. Most importantly, creatine supplementation may have no benefit in patients with creatine transporter defects [103]. Creatine kinase is highly sensitive to oxidative stress due to the presence of cysteine residues, which can be easily modified by reactive oxygen species. It has been revealed that AD patients may have reduced concentrations of phospho-creatine at the onset of the disease and declined oxidative metabolism in advance stages of disease [104]. The use of creatine supplementation in patients with AD remains controversial, despite recent evidence proving its benefits. Functional APP acts as a chaperone targeting mitochondrial creatine kinase and other proteins from cytoplasm to mitochondria, and this chaperoning role could be disrupted by decreased APP function, resulting in decreased levels of mitochondrial creatine kinase, reduced phospho-creatine and thus increased creatine deposits, which support the statement that an intake of creatine supplementation in AD may be futile, leading to a further increase in creatine deposits without bioenergetic improvement [104]. However, creatine supplementation has been shown to improve memory, learning and mental concentration in healthy subjects, suggesting that these effects may be seen in early stages of AD, even though it may not increase cellular bioenergetics in the later stages of the disorder where the creatine kinase system becomes inactivated due to enhanced levels of oxidative reactive species [105,106]. Creatine supplementation may also protect against oxidative stress induced damage to creatine kinase isoenzymes, by preventing the conversion of functional octameric mitochondrial creatine kinase to dimeric mitochondrial creatine kinase, and thus exogenous supplementation with creatine may delay the ROS inactivation of creatine kinase, as normally occurs in AD patients [107]. Another mechanism implicated in the neuroprotection provided by creatine supplementation is the activation of the AMPK signaling pathway, which is essential for regulating mitochondrial contents and functions in a PGC-1α dependent-pathway (which has been shown to be downregulated in post-mortem brain tissues of AD patients and correlates with neurofibrillary tangles) [108,109]. Patients with PD showed an improvement in mood, muscle function and strength in two different clinical studies, when supplemented with creatine [110,111]. Oral administration of creatine demonstrated protection against MTPT induced depletion of dopamine in mice [112], while a phase II clinical trial of creatine in patients in the early stages of PD led Ravina et al. to the statement that creatine supplementation is not futile in these patients [113]. Treatment of HD patients with creatine (10 g/day) for two years resulted in the prevention of weight loss with improvements in neurological testing scores in some patients [114]. A randomized, double-blind, placebo-controlled study showed a significant reduction of 8-hydroxy-2′-deoxyguanosine (an indicator of oxidative damage to DNA) in HD patients when treated with creatine, 8 g/day for 16 weeks [115]. Moreover, creatine showed neuroprotective effects in transgenic models of ALS (*G93A* mice) [116], but no benefits have been seen with creatine intake on the survival and progression of ALS in clinical settings [117,118,119].

CoQ10 (also known as ubiquinone) is a potent antioxidant with electron carrying properties in the mitochondrial respiratory chain, and its deficiency can be associated with five major clinical outcomes, i.e., encephalomyopathy, severe infantile multisystemic disease, cerebellar ataxia, isolated myopathy, and nephrotic syndrome, due to a contribution to respiratory chain defects, increased ROS production and apoptosis [120]. Supplementation with CoQ10 and its analogues (mitoquinone and idebenone) could restore electron flow in mitochondrial respiratory chains and/or improve mitochondrial antioxidant capacity, the most important factors in the management of neurodegenerative disorders [121]. In vivo studies demonstrated the neuroprotective potential of CoQ10 against mitochondrial toxins such as MPTP, malonate, and 3- nitropropionic acid, and increased survival of animals in transgenic mouse models of HD and ALS [122]. It inhibits the activation of mitochondrial membrane permeability transitions, resulting in the reduction of apoptotic processes [123]. Additionally, it also serves as a cofactor for mitochondrial uncoupling proteins, which protect against neuronal damage by decreasing ROS generation [124]. Horvath et al. observed neuroprotection by CoQ10 against MPTP toxicity, facilitated by mitochondrial uncoupling in substantia nigra [125]. Idebenone may recover skeletal and cardiac muscle bioenergetics, and at higher doses may also improve neurological symptoms in patients with FRDA [126]. Intriguingly, mitoquinone (produced by the conjugation of lipophilic triphenylphosphonium cation with a CoQ10 moiety) easily passes mitochondrial membranes due to its positive charge, accumulating several hundred-fold in mitochondria and thus is 800 times more potent an antioxidant than idebenone [126]. 

*G. biloba* extract, containing 24% flavonoids and 6% terpenes, is currently used in clinical practice for the improvement of cognitive dysfunction [127]. In vitro studies showed enhanced A*β* production, improved mitochondrial membrane potential and restored ATP levels in cells with damaged mitochondria [128,129]. Animal studies revealed an improvement in the activities of complexes I, IV and V, and alleviation of nitrosative stress [128]. This neuroprotective mechanism may be attributed to the presence of terpene lactones that acts as ROS scavengers and restore mitochondrial function [128].

Remacemide is a *N*-methyl-d-aspartate (NMDA) receptor antagonist, that synergistically enhanced the neuroprotective effects mediated by CoQ10 in a transgenic mouse model of HD [130]. 

Riluzole is the only drug approved by the Food and Drug Administration (FDA) for the treatment of ALS, which counteracts oxidative stress by induction of glutathione (GSH) production [131]. 

Moreover, α-lipoic acid can be beneficial for age related cognitive decline and peripheral neuropathies via improvement of mitochondrial structure and function, decreasing oxidative damage, elevating antioxidant enzymes and restoration of key enzymes involved in mitochondrial function and redox signaling, such as PGC-1α [132]. 

Omega-3 fatty acids are a sub-group of essential fatty acids with neuroprotective properties, particularly in cognitive dysfunction, and low consumption of these fatty acids may increase age-related cognitive deficits [133]. They offer protection against mitochondrial damage and apoptosis through enhanced MMP and processed non-amyloidogenic amyloid precursor protein which cause the secretion of soluble amyloid precursor protein [134]. Besides, this class of fatty acids also improves the activity of respiratory chain complexes (I and IV), mitochondrial respiration and lipid metabolism [135,136].

## 6. Neuroprotective Potential of *β*-Caryophyllene

Growing evidence suggests that *β*-caryophyllene protects neuronal tissues via the cannabinoid pathway through the activation of CB2 receptors [33,137], which are expressed in various brain regions including cortex, retina, striatum, hippocampus, amygdala, cerebellum, and brainstem, as well as in immune cells and peripheral nervous system pathways involved in the sensation of pain [138]. CB2 receptors are highly expressed in postsynaptic neurons, and activation of these receptors may result in hyperpolarization of the membrane potential, inhibiting the postsynaptic neuron function and thus reducing neuronal excitability [139,140]. In ventral tegmental area (VTA) dopaminergic neurons, these receptors decrease neuronal excitability through modulating K^+^ channel function [139]. CB2 receptors are coupled with the Gq11—phospholipase C—inositol 1,4,5-trisphosphate (PLC—IP3) pathway in prefrontal cortical neurons, which hyperpolarizes the cell membrane through opening of Ca^2+^—dependent Cl^−^ channels [141]. In hippocampal neurons, CB2 receptors activation triggers the stimulation of Na^+^—bicarbonate co-transporter, causing long-term neuronal hyperpolarization [140]. Collectively, these lines suggest the critical impact of CB2 receptors in the mesocorticolimbic system and the potential regulators of the receptors therein, in the regulation of psychiatric and neurobiological activities of the brain.

Collaborating scientists from Spain and Mexico proposed a study to test the link between CB2 receptor activation and neuroprotection by *β*-caryophyllene, involving the exposure of dopaminergic neurons to MPTP which resulted in inactivation of dopaminergic neurons and microglial activation [142]. Treatment of neuronal cells with *β*-caryophyllene prior to MPTP exposure was found to diminish nervous system inactivation and decrease microglial activation. In addition, *β*-caryophyllene also modulates the activity of peroxisome proliferator-activated receptors—gamma (PPAR-γ) and inhibits the activation of toll-like receptors (TLRs), thus reducing the immune-inflammatory pathways in the central and peripheral nervous system [31]. Treatment of male Wistar rats with a *β*-caryophyllene ameliorated high fat/fructose diet induced metabolic and neurobehavioral alterations (insulin resistance, oxidative stress, and neuroinflammation) through activation of PPAR-γ in a ligand dependent manner, via upstream regulation of PGC-1α and CB2 receptor activation [143]. Another study showed an amelioration of Alzheimer-like phenotypes in transgenic (APP/PS1) mice with *β*-caryophyllene, possibly through CB2 receptor activation and a PPAR-γ dependent pathway [144], where the PPAR-γ pathway helps in neuronal survival through the activation of mitogen activated protein kinase kinase/extracellular signal-regulated kinase (MAPKK—½/ERK—½).

Essential oil from *Pterodon emarginatus* Vogel seeds, containing *β*-elemene and *β*-caryophyllene sesquiterpenes, attenuated the neurological signs and the development of autoimmune encephalomyelitis in C57BL/6 mice through the modulation of the Th1/Treg immune balance (decrease in Th1 cell-mediated immune response and upregulation of Treg response in vitro) [145]. The essential oil also inhibited microglial activation and iNOS expression, which may be associated with the inhibition of axonal demyelination and neuronal death in the development of disease. In a murine model of MS, *β*-caryophyllene modulated immune responses by activation of CB2 receptors, reflected by the inhibition of microglial cells, CD4+ and CD8+ T lymphocyte, and protein expression of pro-inflammatory cytokines [146]. *β*-caryophyllene was shown to activate trkA receptors (involved in regulating synaptic strength and plasticity in the nervous system) and induce neuritogenesis in PC12 and SH-SY5Y neuroblastoma cells without affecting nerve growth factor (NGF) or CB2 receptors [147]. In rat primary neurons (in vitro) and rats (in vivo) *β*-caryophyllene improved ischemic brain damage by inhibiting neuroinflammatory response and necroptotic neuronal death, as it decreased mixed lineage kinase domain-like (MLKL), neuronal necrosis, infarct volumes, expression of receptor-interaction protein kinase-1 and 3 (RIPK1 and RIPK3), high-mobility group box 1 (HMGB1), TLR4, tumor necrosis factor-α (TNF-α) and interleukin-1*β* (IL-1*β*) levels [148].

An in vivo study demonstrated the efficacy of *β*-caryophyllene in the treatment of long-lasting, inflammatory, and neuropathic pain in a CB2 receptor dependent manner, as the chronic oral administration of *β*-caryophyllene diminished thermal hyperalgesia and mechanical allodynia with a reduction in spinal neuroinflammation [149]. More interestingly, no tolerability was observed for the analgesic effects of *β*-caryophyllene after prolonged administration. Fotio et al. compared the efficacy of commercially available product Noxiall^®^ (containing *N*-palmitoylethanolamide, *β*-caryophyllene, carnosic acid, and myrrh extract) with gabapentin and pregabalin in a neuropathic pain model of chronic constriction injury using sciatic nerve ligation in mice [150]. A significant attenuation of mechanical allodynia was noted with either Noxiall^®^, gabapentin or pregabalin, and the magnitude of Noxiall^®^ was found comparable to that of gabapentin or pregabalin. Moreover, co-administration of the non-effective doses of Noxiall^®^ and pregabalin significantly decreased neuropathic pain, suggesting additive efficacy. 

## 7. *β*-Caryophyllene: Alteration of Oxidative Stress and Mitochondrial Dysfunction

A number of studies (Table 2) have suggested the alteration of oxidative stress and mitochondrial dysfunction by *β*-caryophyllene and *β*-caryophyllene-containing vegetable extracts, as one of the potential mechanisms in protecting neurons from degeneration [31]. Chávez-Hurtado et al. (2020) observed a reduction in DNA oxidation and overexpression of glial fibrillary acidic proteins with *β*-caryophyllene (10 mg/kg, p.o. for 4 weeks) in the prefrontal cortex and hippocampus of BALB/c mice withd-galactose induced aging [151]. In an in vivo model of PD, *β*-caryophyllene (50 mg/kg, i.p. for 4 weeks) ameliorated oxidative stress (restored antioxidant enzymes, increased GSH, and inhibited lipid peroxidation), neuroinflammation (decreased levels of IL-1*β*, IL-6, and TNF-α, and downregulated COX-2 and iNOS expression), and glial activation as well as rescuing dopaminergic neurons [152]. 

Javed et al. investigated the CB2 receptor mediated neuroprotective effects of *β*-caryophyllene in a rotenone induced animal model of PD [153]. Rotenone (2.5 mg/kg) induced a significant loss in dopaminergic neurons in the substantia nigra pars compacta and dopaminergic striatal fibers, following the activation of astrocytes and microglia when injected peritoneally once daily for 4 weeks. Moreover, rotenone downregulated antioxidant enzymes, increased nitrite levels and induced proinflammatory cytokines (IL-1*β*, IL-6 and TNF-α) and inflammatory mediators (NF-κB, COX-2, and iNOS). Supplementation with *β*-caryophyllene (50 mg/kg once daily for 4 weeks, 30 min prior to rotenone administration) attenuated the induction of pro-inflammatory cytokines and inflammatory mediators, prevented the depletion of glutathione, reduced lipid peroxidation, and augmented antioxidant enzymes (SOD and CAT). Tyrosine hydroxylase immunohistochemistry showed the rescue of dopaminergic neurons and fibers following decreased activation of glial cells.

*β*-caryophyllene protected C6 glioma cells from glutamate induced cytotoxicity through alteration of antioxidant responses, mainly by inhibition of ROS production and restoration of MMP via CB2 receptor dependent nuclear factor erythroid 2–related factor 2 (Nrf2) activation [154]. In a neurovascular unit model of oxygen-glucose deprivation and re-oxygenation—induced injury, *β*-caryophyllene significantly decreased blood–brain barrier (BBB) permeability, reduced neuronal apoptosis, relieved oxidative stress damage, decreased secretion of inflammatory cytokines, downregulated metalloproteinase-9 expression/activity and Bcl-2-associated X protein (Bax) expression, and upregulated expression of claudin-5, occludin, zonula occludens-1 (ZO-1), growth-associated protein-43 (GAP-43) and B-cell lymphoma 2 (Bcl-2) [155]. Conversely, *β*-caryophyllene relieved seizures in mice induced by pentylenetetrazole, but anti-convulsant doses (0, 10, 30, and 100 mg/kg i.p.) showed no benefits over pentylenetetrazole related oxidative stress i.e., thiobarbituric acid-reactive substances and nonprotein thiol content [156].

Lou et al. (2016) found an attenuation of focal cerebral ischemia-reperfusion injury in rats by treatment with *β*-caryophyllene through enhanced expression of Nrf2 and HO-1, and restored activity and expression of antioxidant enzymes, i.e., superoxide dismutase (SOD) and catalase (CAT) [157]. In C57BL/6 mice, *β*-caryophyllene ameliorated the development of experimental autoimmune encephalomyelitis through inhibiting the production of hydrogen peroxide (H2O2), IFN-γ, TNF-α, IL-17 and NO, and decreasing the number of inflammatory infiltrates and neurological damage [158]. An in vitro study demonstrated the alleviation of 1-methyl-4-phenylpyridinium induced neurotoxicity by *β*-caryophyllene through restoring MMP and increasing intracellular activity of GSH and glutathione peroxidase (GPx), where antioxidant effects were found to be CB2 receptor dependent [159]. Apoptosis was prevented in same study by inhibition of the up-regulation of caspase-3 and Bax, restoring Bcl-2 expression, and suppressing heme oxygenase-1 (HO-1) activation and c-Jun *N*-terminal kinase (JNK) phosphorylation.

*Pinus halepensis* Mill. essential oil attenuated Alzheimer’s toxic A*β* (1-42)-induced memory impairment and oxidative stress in rat hippocampus [160]. Inhalation of *P. halepensis* essential oil (1 and 3%) for 21 days resulted in the inhibition of hippocampal AChE activity, elevation of hippocampal antioxidant markers (SOD, CAT, GPx and GSH), and attenuation of A*β*-induced elevation of malondialdehyde (MDA) levels. Phytochemical screening of essential oils revealed the presence of 45 different compounds, with 33 of those compounds having been identified and quantified. Sesquiterpenes (*β*-caryophyllene) and monoterpenes (α-pinene, myrcene, terpinolene, and 2-phenylethylisovalerate) were the most abundant compounds present in the essential oil, while diterpenes (mainly cembrene) represent only 2.50% of the phytochemicals present. More importantly, *β*-caryophyllene was found to be the most abundant compound present with the highest percentage of 29.45%.

Essential oils extracted from the dried leaves of *Aloysia citrodora* Palau displayed significant antioxidant and protective effects against both H_2_O_2_ and A*β*-induced neurotoxicity in CAD neuroblastoma cell lines [161]. H_2_O_2_ (250 μM) and A*β* (10 μM) failed to elicit neurotoxic responses in the presence of *A. citrodora* essential oil (0.01 and 0.001 mg/mL). The in vitro antioxidant effects of *A. citrodora* essential oil was confirmed by its Fe^2+^ chelating capacity. The major chemical components detected in this essential oil were limonene, geranial, neral, 1, 8-cineole, curcumene, spathulenol and caryophyllene oxide. 

The clove oil obtained from *Syzygium aromaticum* (L.) Merr. and L.M. Perry is known to contain eugenol as its most abundant compound (87.34%), with eugenol acetate (5.18%) and *β*-caryophyllene (2.01%) being present in smaller amounts [162]. Kumar et al. reported the neuroprotective potential of clove oil in intra-cerebroventricular (ICV) colchicine-induced memory impairment in rats [163]. Treatment of colchicine challenged rats with *S. aromaticum* (0.05 mL/kg and 0.1 mL/kg, i.p.) significantly improved cognitive dysfunction, with a marked reduction of AChE activity, lipid peroxidation levels, and nitrite concentrations, and restoration of GSH and mitochondrial respiratory enzyme complex (I–IV) activities. Authors linked the attenuation of cognitive dysfunction with antioxidant and mitochondrial restoring mechanisms. *Hyptis fruticosa* Salzm. ex Benth (also known as *Eplingiella fruticosa*) leaf essential oil (containing *β*-caryophyllene, bicyclogermacrene and 1,8-cineole), complexed with *β*-cyclodextrin, showed neuroprotective effects in a mouse model of PD by decreasing membrane lipid peroxide levels in the striatum and preserving dopaminergic depletion in the striatum and substantia nigra pars compacta, when administered at a dose of 5 mg/kg, p.o. for 40 days [164]. 

*Ocimum basilicum* L. essential oil attenuated ethidium bromide-induced cognitive deficits as well as neuroinflammation, astrogliosis and mitochondrial dysfunction in the prefrontal cortex of rats, with induced MS like manifestations [165]. *O. basilicum* (100 and 200 μL/kg) significantly mitigated ethidium bromide-induced neuroinflammation by increasing the levels of proinflammatory cytokines (TNF-α and IL-6) and astrogliosis by increasing (Glial fibrillary acidic protein (GFAP) and Ionized calcium binding adaptor molecule-1 (Iba-1) levels. In addition, mitochondrial function, integrity, respiratory control rate, ATP production, and mitochondria-dependent apoptosis were positively regulated in the prefrontal cortex of rats by treatment with *O. basilicum*. Chemical analysis of the essential oil derived from *O. basilicum* L. demonstrated the presences of several phytoconstituents, with methyl chavicol, geranial, neral and caryophyllene oxide being major components [166]. *Salvia rosmarinus* Spenn. essential oil (comprised chemically of 1,8-cineole, α-pinene, camphor, and trans-caryophyllene) exhibited strong antioxidant effects evaluated by DPPH, ABTS, FRAP and *β*-carotene bleaching tests and confirmed by the relative antioxidant capacity index and significant acetylcholinesterase (AChE) inhibitory activities, suggesting neuroprotective potential in patients with AD [167].

## 8. Toxicological Aspects

According to the opinion published by the Scientific Panel on Food Additives, Flavorings, Processing Aids and Materials in Contact with Food, aimed at advising the European Commission on the implications for human health of chemically defined flavoring substances used in or on foodstuffs in the Member States, *β*-caryophyllene did not show any mutagenicity [168]. Acute toxicity studies have revealed no signs of toxicity in female Swiss mice up to 2000 mg/kg dose, whereas the median lethal dose (LD_50_) has been calculated as doses greater than 5000 mg/kg [168]. Sub-chronic toxicity evaluation showed no signs of adverse effects in Wistar rats with the administration of 700 mg/kg/day *β*-caryophyllene for 90 consecutive days [169]. Another study demonstrated the absence of adverse clinical signs and mortality in female Swiss mice with single and repeated doses of oral *β*-caryophyllene. No considerable changes were observed in term of body weight, food/water intake, oxidative stress markers, and other hematological and biochemical parameters [169,170]. Moreover, no signs of neurotoxicity have been noted in rodents at the dosages usually used for evaluating pharmacological effects, i.e., 20–100 mg/kg [171]. 

Moreover, Molina-Jasso et al. while investigating clastogenicity of *β*-caryophyllene, did not observe any signs of genotoxicity or bone marrow cytotoxicity in mice [172]. Although, in vivo studies confirmed *β*-caryophyllene as a safe agent, general consideration of possible drug interactions should be taken into account [173]. A research conducted by Nguyen et al. involving subcellular fractions of hepatic tissues of rats and humans, demonstrated inhibition by *β*-caryophyllene and *β*-caryophyllene oxide on the activities of the enzymes involved in the metabolism and detoxification of xenobiotics, i.e., cytochrome p450 isoforms (CYP1A2, CYP3A4, CYP2A6, CYP2B6, CYP2C9, CYP2C19, CYP2D6, and CYP2E1). More pronounced effects were noted against CYP1A2 and CYP3A4. It is evident that the inhibition of enzymes may considerably enhance drug levels in the body, with prolonged duration of action and increased toxicity [173].

## 9. Concluding Remarks

Oxidative stress and mitochondrial dysfunction are well organized events in the degeneration of neuronal cells. Targeting mitochondria in chronic degenerative disorders is not a novel idea. Many promising experimental therapeutics are known to enhance mitochondrial function, unfortunately none have yet proven successful in halting the development and progression of neurodegeneration. Literature data suggest that *β*-caryophyllene, a dietary phytocannabinoid, possesses a neuroprotective capability through decreasing oxidative stress and stabilizing mitochondria, and could be a potential lead molecule in the discovery of drugs for neurodegenerative disorders. Besides CB2 receptor agonism, *β*-caryophyllene has been found to positively regulate PPAR-γ, TLRs and neuroimmune pathways, as possible targets implicated in the protection against neuronal loss. Essential oils containing *β*-caryophyllene, extracted from different vegetable sources, also showed promising neuroprotective effects following their attenuation of oxidative stress and/or mitochondrial dysfunction. However, it remained unknown whether these beneficial effects could be attributed to the summation of the activities of phytoconstituents present in essential oils, or to a single compound mediating the observed effects. Nevertheless, the available data are not sufficient to draw any clinical conclusion for the recommendation of *β*-caryophyllene in the management of neurodegenerative disorders, and an expansion of the literature is strongly needed, in particular regarding the most effective doses for beneficial roles of *β*-caryophyllene in the management of neurodegenerative disorders, and the potential benefits of *β*-caryophyllene in targeting mitochondria in neurodegenerative diseases utilizing both experimental and human studies.

## Figures and Tables

**Table 1:** Vegetable sources of *β*-caryophyllene and percentage composition of their essential oils (data extracted from the Essential Oil Database) [27].

Botanical Name	Family	Active Parts	Percentage ^1^
*Ocimum basilicum* L.	Lamiaceae	Leaf	0.3–3.1
*Cinnamomum* species	Lauraceae	Leaf/bark ^a^	0.2–35.9 ^a^
*Piper nigrum* L.	Piperaceae	Berries/Leaf/stem ^b^	3.3–46 ^b^
*Syzygium aromaticum* (L.) Merr. and L.M. Perry	Myrtaceae	Floral bud	3.2
*Cannabis sativa* L.	Cannabaceae	Whole plant (fresh material)	3–16.2
*Lavandula angustifolia* Mill.	Lamiaceae/labiatae	Flower and stem	1.08
*Lavandula angustifolia* Mill.	Lamiaceae/labiatae	Whole plant	0.3
*Origanum vulgare* L.	Lamiaceae/labiatae	Leaf/Stem/Flower/Whole plant^c^	0.4–24.5 ^c^
*Rosmarinus officinalis* L.	Lamiaceae	Aerial parts	0.5–13.6

^1^ Percentage of the compound calculated by comparing the gas chromatographic peak area of the analyte with the total area of all detected peaks. ^a^ Depending upon different species. ^b^ Depending upon different cultivars. ^c^ Depending upon sub-species.

**Table 2:** Alteration of oxidative stress and mitochondrial dysfunction by *β*-caryophyllene and vegetable extracts containing *β*-caryophyllene.

Study Model	Extract or Compound (Dose/Concentration)	Study Outcomes	References
BALB/c mice, with d-galactose induced aging	*β*-caryophyllene (10 mg/kg/day, p.o. for 4 weeks)	↓ DNA oxidation and overexpression of glial fibrillary acidic proteins in the prefrontal cortex and hippocampus.	[151]
Rats, with PD	*β*-caryophyllene (50 mg/kg/day, i.p. for 4 weeks)	↑ GSH, SOD and CAT. Inhibit lipid peroxidation. ↓ IL-1*β*, IL-6, and TNF-α levels. ↓ COX-2 and iNOS expression. ↓ glial activation and rescued dopaminergic neurons.	[152]
Rats with PD	*β*-caryophyllene (50 mg/kg/day, i.p.) for 4 weeks	↓ pro-inflammatory cytokines (IL-1*β*, IL-6 and TNF-α) and inflammatory mediators (NF-κB, COX-2, and iNOS). ↑ glutathione, SOD and CAT. ↓ lipid peroxidation.	[153]
C6 glioma cell line	*β*-caryophyllene (0.5 and 1.0 μM)	↑ cellular antioxidant responses via CB2 receptor dependent Nrf2 activation. ↓ ROS production. Restored MMP.	[154]
Neurovascular unit (BMECs, neurons and astrocytes)	*β*-caryophyllene (10 μmol/L)	↓ BBB permeability, neuronal apoptosis, oxidative stress damage, inflammatory cytokines. ↓ metalloproteinase-9 expression/activity, Bax expression. ↑ expression of claudin-5, occludin, ZO-1, GAP-43, and Bcl-2.	[155]
Adult male Sprague–Dawley rats, with focal cerebral ischemia	*β*-caryophyllene (34, 102 and 306 mg/kg/day, p.o.).	↑ Nrf2 and HO-1 expression. Restored SOD and CAT activity and expression.	[157]
C57BL/6 mice, with autoimmune encephalomyelitis	*β*-caryophyllene (25 and 50 mg/kg/day, p.o.).	↓ H_2_O_2_, IFN-γ, TNF-α, IL-17 and NO.	[158]
Human neuroblastoma SH-SY5Y cells	*β*-caryophyllene (1 and 2.5 μM)	Restored reduction in MMP. ↑ intracellular GSH and GPx activity. ↓ Caspase-3 and Bax.Restored Bcl-2 expression Suppressed HO-1 activation and JNK phosphorylation.	[159]
Wistar rats-male with A*β* (1-42)-induced memory impairment	*Pinus halepensis* essential oil (1 and 3%).	↓ hippocampal AChE activity. ↑ hippocampal antioxidant markers (SOD, CAT, GPx and GSH). ↓ malondialdehyde (MDA) levels.	[160]
CAD neuroblastoma cell lines	*Aloysia citrodora* Palau essential oil (0.01 and 0.001 mg/mL)	↓ H_2_O_2_ (250 μM) and A*β* (10 μM) induced neurotoxicity. Fe^2+^ chelation in vitro.	[161]
Rats, with ICV colchicine induced memory impairment	*Syzygium aromaticum* (L.) Merr. and L.M. Perry (0.05 mL/kg and 0.1 mL/kg)	↓ AChE activity, lipid peroxidation levels, and nitrite concentrations. Restored activities of GSH and mitochondrial respiratory enzyme complex (I–IV).	[163]
Male mice, with PD	*Eplingiella fruticosa* leaf essential oil. (5 mg/kg/day, p.o. for 40 days)	↓ membrane lipid peroxide levels in the striatum. ↑ dopamine levels in the striatum and substantia nigra pars compacta.	[164]
Male adult Wistar albino rats, with induced MS like manifestations	*Ocimum basilicum* L. essential oil (100 and 200 μL/kg)	↓ proinflammatory cytokines (TNF-α and IL-6) in prefrontal cortex. ↓ astrogliosis by increasing GFAP and Iba-1 levels in prefrontal cortex. ↑ mitochondrial function, integrity, respiratory control rate and ATP production. ↓ mitochondria-dependent apoptosis in prefrontal cortex of rats.	[165]
In vitro, antioxidant and AChE inhibition assays	*Salvia rosmarinus* Spenn. essential oil.	Strong antioxidant effects (DPPH, ABTS, FRAP and *β*-carotene bleaching tests). Significant AChE inhibition.	[167]

AChE, acetylcholinesterase; Bax, Bcl-2-associated X protein; BBB, blood–brain barrier; Bcl-2, B-cell lymphoma 2; BMECs, brain microvascular endothelial cells; CAT, catalase; CB2 receptor, cannabinoid-2 receptor; GAP-43, growth-associated protein-43; GFAP, Glial fibrillary acidic protein; GPx, glutathione peroxidase; GSH, glutathione; H_2_O_2_, hydrogen peroxide; HO-1, heme oxygenase-1; Iba-1, ionized calcium binding adaptor molecule-1; IL-1*β*, interleukin-1*β*; IL-6, interleukin-6; iNOS, inducible nitric oxide synthase; JNK, c-Jun *N*-terminal kinase; MDA, malondialdehyde; MMP, mitochondrial membrane potential; Nrf2, nuclear factor erythroid 2–related factor 2; ROS, reactive oxygen species; SOD, superoxide dismutase; TNF-α, tumor necrosis factor- α; ZO-1, zonula occludens-1; “↓” reduction; “↑”increment.
